# Effect of Remote Tai Chi Training vs. Strength Training on Muscle Mass and Grip Strength in Older Mexican Adults in the Context of the COVID-19 Pandemic

**DOI:** 10.3390/sports14090372

**Published:** 2026-09-01

**Authors:** Nayeli Vaquero-Barbosa, Lilia Castillo-Martínez, Juan Garduño-Espinosa, Jimena Aguilar-Curiel, Graciela Gavia-García, Cristina Flores-Bello, Elsa Correa-Muñoz, Víctor Manuel Mendoza-Núñez

**Affiliations:** 1 Research Unit on Gerontology (UIG), FES Zaragoza, National Autonomous University of Mexico (UNAM), Mexico City 09230, Mexico; naye7293@gmail.com (N.V.-B.); jime_v17@hotmail.com (J.A.-C.); ggg1501@hotmail.com (G.G.-G.); rasguosaflores@yahoo.com.mx (C.F.-B.); elsa.correa@zaragoza.unam.mx (E.C.-M.); 2 Postgraduate Master and Doctorate in Health Sciences, National Autonomous University of Mexico (UNAM), Mexico City 04510, Mexico; 3 National Institute of Medical Sciences and Nutrition Salvador Zubirán (INCMNSZ), Mexico City 14080, Mexico; cam7125@gmail.com; 4 Federico Gómez Children’s Hospital of Mexico (HIMFG), Mexico City 06720, Mexico; juan.gardunoe@gmail.com

**Keywords:** tele-exercise, tai chi, strength training, older adults, body composition, handgrip strength

## Abstract

Tele-exercise (TEF) is an option for maintaining the vitality of people who have difficulty exercising outside the home and in confinement situations. The aim of the present study was to determine the effect of TEF in Tai Chi (TC) compared to Strength Training (ST) on muscle mass and strength in older Mexican adults. The effect was measured in the following groups: (i) TC Group (TCG) n = 22; (ii) ST Group (STG) n = 22; and (iii) Control Group (CG) n = 21. Skeletal muscle mass (SMM), Skeletal Muscle Mass Index (SMMI), FM (%) (percentage of fat mass), handgrip strength (HS), and sit–stand test (SST) were assessed before and after the intervention. The data presented comes from a per-protocol design and only include the results of people who completed the study. An ANCOVA was performed and the mean difference (MD) was estimated with a 95% confidence interval (CI). The effect size was also calculated using the partial squared eta (η^2^p). A high percentage of study dropout was observed in all three groups (TCG, 20/42 = 48%; STG, 19/41 = 46%; CG, 19/40 = 47%). Both TCG and STG showed an increase respect CG in SMM (TCG: MD = 1.79, CI 0.71, 2.88; STG: MD = 1.10, CI 0.18, 2.02, *p* < 0.01) and SMMI (TCG: MD = 0.73, CI 0.28, 1.19; STG: MD = 0.40, CI 0.05, 0.77, *p* < 0.01). Likewise, they showed a significant decrease in FM (%) (TCG: MD −4.81, CI −8.17, −1.44; STG: MD = −3.99, CI −6.84, −1.13, *p* < 0.01). Regarding the effect size, it was observed large statistically significant effects in SSM (η^2^p = 0.19, F = 5.38, *p* < 0.01); SMMI (η^2^p = 0.19, F = 5.23, *p* < 0.01); FM% (η^2^p = 0.18, F = 5.06, *p* < 0.01); FM/FFM (η^2^p = 0.19, F = 5.47, *p* < 0.01); and STST (η^2^p = 0.16, F = 4.42, *p* < 0.01). Our findings suggest that Tai Chi or Strength Training tele-exercise has a similar positive effect on skeletal muscle mass coupled with a decrease in the percentage of fat mass, so one of these exercises could be an option to continue with physical exercise training at home for older adults in confinement situations. However, the results cannot be generalized due to limitations in the quasi-experimental study design, the non-representative sample size, the high dropout rate, and selection bias. For this reason, more randomized intention-to-treat clinical trials with representative samples are necessary.

## 1. Introduction

When the first case of coronavirus disease (COVID-19) was reported in Wuhan Province, China, in late 2019, no one could have predicted that we were facing one of the greatest health crises of our time [1]. Given that this disease is transmitted from person to person through respiratory droplets, one of the measures taken to slow its rapid spread was social isolation to reduce the rate of infection and mortality associated with the SARS-CoV-2 virus, especially in vulnerable groups such as people over 65 years of age, those with obesity, smokers, those with more than one chronic disease (diabetes and hypertension, among others), those who are physically inactive, or those with low lung capacity [1,2,3]. For this reason, it is essential to propose alternatives to continue physical exercise programs, maintain a healthy diet and functional capacity, and avoid social isolation, since COVID-19 redefined the risk of frailty in older adults due to the increase in inflammation, oxidative stress, and a higher incidence of sarcopenia, neurological problems, and other comorbidities [4,5]. In this regard, some systematic reviews have reported that synchronous tele-exercise and guided te le-rehabilitation are feasible, safe, and well-accepted by older adults, with high adherence rates and clinically significant improvements in muscle strength, mobility, balance, physical performance, and functional capacity [6,7,8]. Furthermore, real-time supervision via videoconferencing platforms allows for immediate feedback, improves exercise execution, and increases participant safety, supporting tele-exercise as a valid alternative when face-to-face programs are not feasible [9].

However, in our context, several challenges exist in the use of this technology, such as low levels of education, lack of training, fear and negative attitudes toward technology, insecurity regarding the use of personal data, limited economic resources or inadequate infrastructure, and inappropriate applications for older adults [10,11]. In this sense, population groups with access to this resource could benefit from this approach, as positive results have been reported through digital health or telehealth interventions [12]. Therefore, one of the priority strategies for maintaining regular physical exercise and avoiding social isolation, especially in confinement situations such as that experienced during the COVID-19 pandemic, is the implementation of structured and supervised tele-exercise programs.

The most recommended types of physical exercise in old age, especially to prevent or limit the progression of sarcopenia, are Strength Training (ST) and Tai Chi (TC). In this sense, a systematic review that included fourteen randomized controlled trials (RCTs) on the effect of ST on skeletal muscle mass (SMM), grip strength, and physical performance in older adults with sarcopenia without comorbidity found a statistically significant positive effect on all variables except SMM [13]. Likewise, Yang et al. (2025), in a systematic review conducted in older adults with sarcopenia that included eleven randomized controlled trials (RCTs), reported a statistically significant positive effect after cardiopulmonary exercise training on gait speed, muscle strength, and grip strength; however, they did not find significant changes in SMM [14].

On the other hand, Tai Chi, by combining slow, multidirectional movements, postural control, weight transfer, balance, coordination, respiratory control, and cognitive attention, in addition to its effects on muscle strength, simultaneously improves balance, neuromuscular control, mobility, and functional performance, making it especially suitable for older adults and for home-based interventions that require minimal equipment and a lower risk of injury [15,16].

Within this framework, it is hypothesized that tele-training of TC will have a greater or similar effect than ST on skeletal muscle mass and grip strength. Therefore, the purpose of the present study was to determine the effect of tele-training of TC compared to ST on these parameters in the context of the COVID-19 pandemic.

## 2. Materials and Methods

### 2.1. Participants and Design

After obtaining informed consent, a quasi-experimental study was conducted in a convenience sample, following the guidelines of TREND (Transparent Reporting of Evaluations with Non-Randomized Designs) [17]. The protocol was approved by the ethics committee of the Faculty of Higher Studies Zaragoza, UNAM (FESZ/DEPI/CI/03920) and registered on a clinical trials platform (ISRCTN48485253) [18].

The sample size was estimated a priori using G*Power version 3.1.9.7 (Heinrich Heine University of Düsseldorf, Germany) for a repeated-measures ANOVA with one within-between-group, considering three study groups and two measurement points (baseline and 6 months). Skeletal muscle mass was selected as the primary outcome. Assuming a moderate effect size (f = 0.25), a significance level of α = 0.05, statistical power of 80%, a correlation of 0.50 between repeated measures, and a non-sphericity correction of ε = 1.0, the required sample size was 126 participants (42 per group). Assuming a dropout rate of 20%, the recruitment target increased to 153 participants (51 per group). However, due to the emerging pandemic situation, it was only possible to complete a sample of 123 adults aged 60 to 74 years from Mexico City who had a stable Wi-Fi connection and a device for making video calls, without severe cognitive impairment (Montreal Cognitive Assessment, MoCA score ≥23), and who did not engage in regular physical exercise in the last 6 months (<60 min per week), without risk of sarcopenia assessed through the SARC-F screening test, considering as the cutoff point ≤3 [19], nor a history of hospitalization in the last 6 months.

The exercise groups were randomly assigned by clusters, defined as geographical groupings within Mexico City and its surroundings. Six clusters were identified and randomized using randomizer.org. Each cluster was assigned to one of the exercise interventions: Tai Chi (n = 42) or Strength Training (n = 41). The Control Group (n = 40) was self-selected and consisted of individuals who, for personal reasons, chose not to participate in the exercise program but agreed to participate in weekly remote meetings to share their experiences during lockdown.

**Figure 1:**
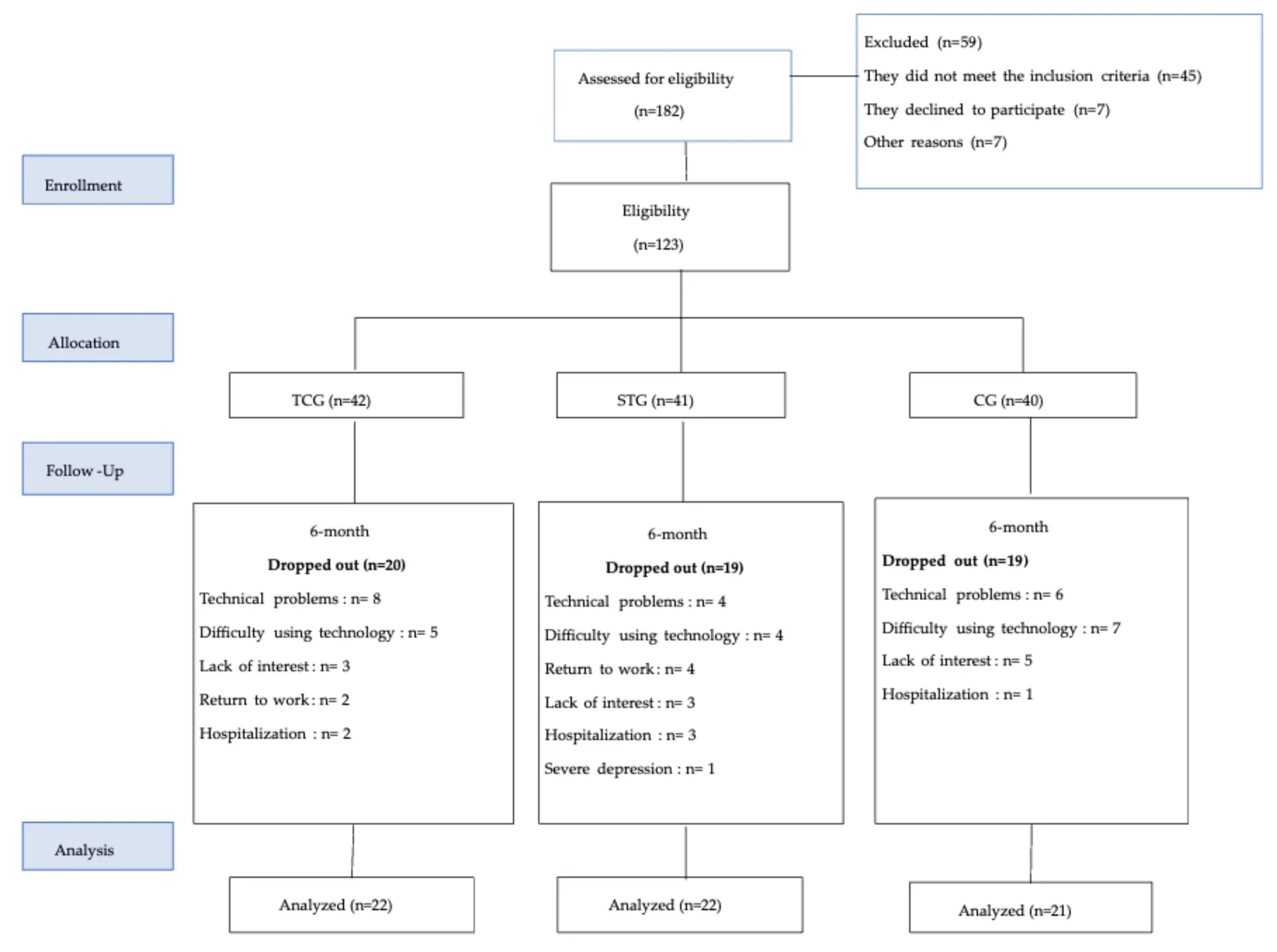
Flowchart of the participant tracking process.

The TCG and STG participated in a formal exercise training program for six months (4 days/week/60 min) supervised by trained instructors. The CG participated in weekly 60 min meetings coordinated by a member of the research team. During the intervention, several participants dropped out of the study for different reasons (TCG n = 20; STG n = 19; and CG n = 19) (Figure 1).

An initial interview was conducted with all participants to collect sociodemographic characteristics and clinical data. They were also assessed before and six months after the intervention on anthropometric parameters, dietary intake, body composition, grip strength, and physical performance.

### 2.2. Anthropometric Measurements

To determine the patients’ body dimensions, a calibrated medical scale (SECA^®^, Hamburg, Germany) was used for body weight and a wall-mounted stadiometer (SECA^®^, Hamburg, Germany) for height. Patients were asked to stand with their heels together, their head erect in the Frankfort plane and in contact with the stadiometer. Both measurements were used to calculate BMI using the formula, weight (kilograms)/height^2^ (meters), following the standardized criteria established by the Ministry of Health of Mexico [20]. All measurements were performed by trained researchers from our working group.

### 2.3. Dietary Intake

Dietary intake was determined using the 24 h recall technique of a typical weekday and a weekend day, performed by trained nutritionists. To ensure portion sizes, household sample measures (cups and spoons) were used [21]. Analysis was subsequently performed using FoodProcessor^®^ 2016 software.

### 2.4. Body Composition

Previously trained personnel performed all measurements in the morning using the same bioimpedance analyzer, following a standardized protocol and under similar environmental conditions. Before measuring body composition using bioelectrical impedance analysis (BIA), researchers were trained to perform standardized measurements. Participants also received written and verbal instructions to minimize factors that could affect the BIA measurement. They were instructed to fast overnight (8 to 12 h), maintain their usual hydration level, avoid strenuous physical activity for 24 h prior to the assessment, abstain from alcohol and caffeine, empty their bladder immediately before the measurement, and continue their regular medication.

Bioelectrical impedance analysis (BIA) was performed to assess muscle mass using a four-pole, single-frequency device (50 kHz, Quantum X, RJL System^®^ Clinton, Township, Míchigan,USA). One pair of electrodes was placed on the back of the hand and another pair on the foot on the right side of the body to record resistance and reactance.

Skeletal muscle mass (SMM) was estimated using the Janssen equation [22,23].

$$SSM(\mathrm{kg})=[(\mathrm{height}\;\mathrm{in}\;cm^{2}/\;\mathrm{Resistence})\times 0.401]+(\mathrm{sex}\times 3.825)+(\mathrm{age}\times -0.071)+5.102$$

where sex (males = 1 or females = 0) and age are measured in years.

The Skeletal Muscle Mass Index (SMMI) was estimated with the following equation.

$$SMMI(kg/m^{2})=\frac{\mathrm{SMM}\;\mathrm{in}\;\mathrm{kg}}{\mathrm{height}\;\mathrm{in}\;m^{2}}$$

Subsequently, the fat-free mass (FFM), percentage of FFM (% FFM) and percentage of fat mass (%FM) were calculated as follows [23]:

FFM (kg) = 0.7374 × (Ht2/R) + 0.1763 × (BW) − 0.1773 × (Age) + 0.1198 × (Xc) − 2.4658

where Ht2 is height in cm, R is resistance in ohms, BW is body weight in kg, age is in years, and Xc is reactance in ohms:

$$\%FFM\;=\;\frac{\left(FFM\times 100\right)}{\mathrm{weigtht}\;\mathrm{in}\;\mathrm{kg}}$$

%FM=100−%FFM

### 2.5. Grip Strength

To measure grip strength, a Jamar hydraulic dynamometer, adjustable to hand width, with a measurement range of 0 to 100 kg, was used. The participant had to stand with their arms extended parallel to their torso, holding the dynamometer in their hand, and, without support, was asked to exert maximum force. Three alternating measurements were obtained for each arm, with a one-minute rest between each. The value of each measurement was recorded, and the maximum value was considered [24,25].

### 2.6. Phase Angle (PA)

The PA was estimated using the following formula [26]:

PhA = arctangent (reactance/resistance) × (180°/π)

### 2.7. Physical Performance

Physical performance was assessed using the Short Physical Performance Battery (SPPB), which evaluates balance, gait, strength, and endurance. This test consists of three subtests: (i) balance in three positions: feet together, semi-tandem, and tandem, each held for 10 s; (ii) lower limb strength: time required to stand up from and sit down from a chair five consecutive times, as quickly as possible; and (iii) gait speed: time taken to walk 4 m at a normal pace. The total score classifies physical performance as low (≤8 points) or high (≥9 points) [27].

### 2.8. Physical Tele-Exercise Program

**Figure 2:**
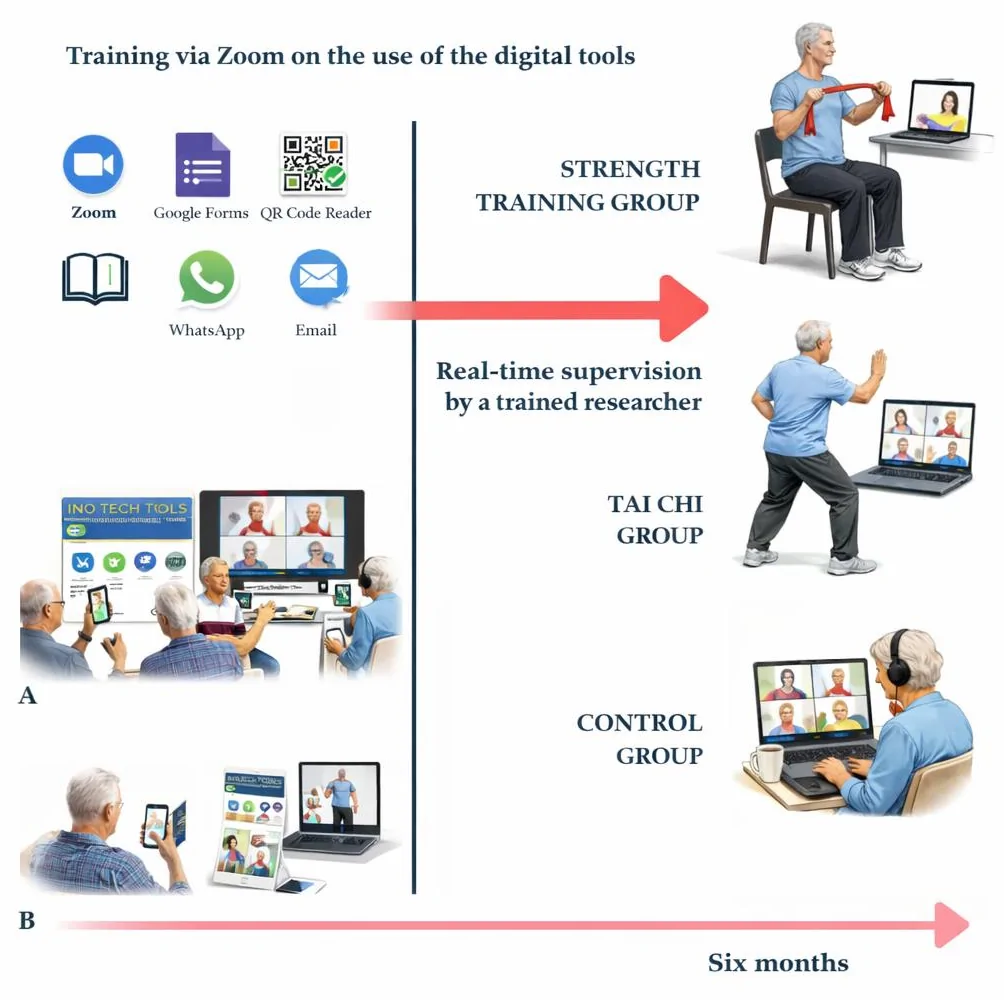
Synchronous real-time tele-intervention. (**A**) Training on the use of digital devices. (**B**) Real-time training and supervision.

Prior to the implementation of the physical tele-exercise program, participating older adults received training via Zoom on the use of the digital tools that would be used in the program, such as: (i) use of computers, tablets, and/or smartphones; (ii) use of Zoom; (iii) Google Forms and QR code readers; and (iv) use of WhatsApp and email. They were provided with a manual and a video. The program was implemented synchronously with real-time supervision by a trained researcher. The first stage involved training on the use of digital devices, followed by continuous supervised training (Figure 2).

The instructors were standardized to deliver the tele-exercise training by specialists from the Department of Sports Activities at the Faculty of Health Sciences Zaragoza, UNAM. All interventions were conducted online with the support of the Zoom application; for the exercise groups, the Borg scale of perceived exertion was used.

The intensity of the exercise was maintained at a light to moderate level (11–13 on the Borg scale of perceived exertion), and the instructors adjusted the speed and complexity of the movements according to the participants’ functional capacity.

#### 2.8.1. Tai Chi Training

The remote Tai Chi training program followed the guidelines established by Li et al. (2003) for the practice of “Eight Forms Tai Chi for Older Adults” [28], which was conducted remotely and supervised via Zoom four days a week, in 60 min sessions for 6 months. Each session consisted of a 10 min warm-up with joint mobility and breathing exercises, followed by 40 min of practice of the Eight-Movement Tai Chi Form, which included weight shifts, multidirectional steps, postural control, coordinated upper and lower limb movements, trunk rotation, balance, breath regulation, and movement sequencing. The session concluded with a 10 min cool-down with stretching and breathing exercises (Supplementary Table S1).

#### 2.8.2. Strength Training

Participants completed a Strength Training program designed by the Gerontology Research Unit, consisting of three phases—(i) warm-up and activation (10 min), (ii) main session (40 min), and (iii) cool-down (10 min)—four sessions per week (WHO, 2020) [29]. The intensity ranged from light to moderate according to the Borg scale. The program utilized progressive overload: the first three months focused on bodyweight exercises, followed by external loads using resistance bands (Theraband) and dumbbells. Each session consisted of three exercise cycles of three sets of 8 to 12 repetitions, with one minute of rest between sets. Exercises included wall push-ups, wall walks, bicep curls, and others, with increasing complexity and load progressively. Participants performed their routines remotely and under the supervision of a qualified instructor via Zoom (Supplementary Table S2).

#### 2.8.3. Control Group

Weekly 60 min meetings were scheduled in which the participants of this group shared their experiences of the COVID-19 pandemic, and the coordinators also gave some talks on healthy aging.

### 2.9. Statistical Analysis

The data presented correspond to a per-protocol design. Statistical significance was set at *p* < 0.05 for all tests using SPSS version 25. The normality of continuous variables was assessed using the Shapiro–Wilk test. Normally distributed variables are presented as mean ± standard deviation (SD), whereas non-normally distributed variables are expressed as median and interquartile range (IQR; P25–P75). Categorical variables are summarized as frequencies and percentages.

Baseline characteristics among the three study groups were compared using one-way analysis of variance (ANOVA) for normally distributed variables and the Kruskal–Wallis test for non-normally distributed variables. Categorical variables were compared using the chi-square (χ^2^) test.

Within-group changes over time for non-normally distributed variables were analyzed using the Friedman test. When a significant overall effect was detected, pairwise comparisons were performed using the Wilcoxon signed-rank test with Bonferroni correction to account for multiple comparisons.

To evaluate the effect of the intervention on the outcome variables at the end of the study, an analysis of covariance (ANCOVA) was performed. This model was adjusted for the presence of Type 2 Diabetes Mellitus, gender, years of schooling, number of household members, and Systolic and Diastolic Blood Pressure (SBP and DBP). Prior to this, the assumption of homogeneity of regression slopes was verified by assessing the interaction between groups and covariates, and homogeneity of variances was assessed using Levene’s test. The analysis showed that this interaction was not statistically significant. When significant overall differences were observed, post hoc pairwise comparisons were conducted using the Bonferroni correction. In addition, the adjusted mean difference (MD) between groups was estimated with its corresponding 95% confidence interval (95% CI) comparing TCG vs. CG, STG vs. CG and TCG vs. STG.

In addition, the percentage change from baseline was calculated using the following formula:

Δ% = [(final value − baseline value)/baseline value] × 100

Partial Eta Squared (*η*^2^_*p*_) was also estimated:

$${\eta^{2}}_{p}=\frac{SS\;effect}{SS\;effect+SS\;error}$$

*SS effect* is the sum of squares of the factor.

*SS error* is the sum of squares of the residual error of the model.

Partial Eta Squared value: (i) close to ≥0.01 is considered low, (ii) ≥0.06 medium, and greater than ≥0.14 large [30].

## 3. Results

The data presented comes from a per-protocol design and only includes the results of people who completed the study. In this regard, a high percentage of study dropout was observed in all three groups (TCG, 20/42 = 48%; STG, 19/41 = 46%; CG, 19/40 = 47%); the reasons are indicated in Figure 1. The sociodemographic characteristics and health status of the study population are presented in Table 1.

### 3.1. Nutrition

After six months of intervention, no statistically significant changes in dietary intake were observed among the three groups (*p* > 0.05) (Table 2).

### 3.2. Body Composition

TCG and STG showed a statistically significant changes in comparison to the CG in SMM (TCG: MD = 1.79, CI 0.71, 2.88; STG: MD = 1.10, CI 0.18, 2.02, *p* < 0.01); SMMI (TCG: MD = 0.73, CI 0.28, 1.19; STG: MD = 0.40, CI 0.05, 0.77, *p* < 0.01); FM (%) (TCG: MD = −4.81, CI, −8.17, −1.44; STG: MD = −3.99, CI −6.84,−1.13, *p* < 0.01); and FM/FFM (TCG: MD = −0.14, CI −0.23, −0.54; STG: MD = −0.13, CI −0.20, −0.05, *p* < 0.01) (Table 3).

### 3.3. Physical Performance and Grip Strength

The overall SPPB sub-test score showed an increase, although only it was observed a change statistically significant in STST time in TCG (MD = −4.47, CI −6.90, −2.04) and STG (MD = −2.02, CI −4.07, −0.04) compared to CG (MD = 1.21, CI −1.78, 4.20) (Table 4).

### 3.4. Percentage Change After Intervention

**Figure 3:**
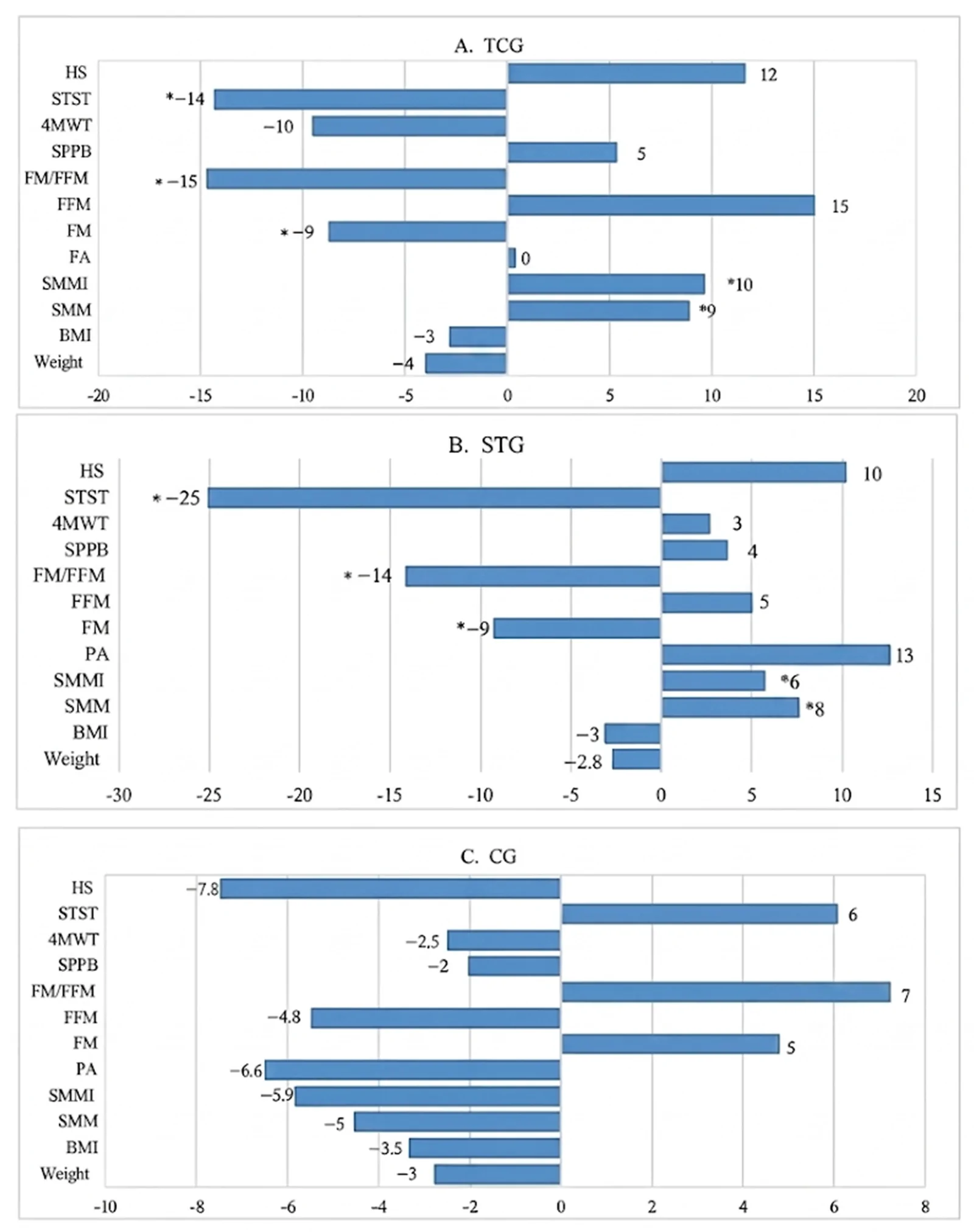
Abbreviations: BMI, Body Mass Index; SMM, skeletal muscle mass; SMMI, Skeletal Muscle Mass Index; PA, phase angle; FM, percentage of fat mass; FFM, fat-free mass; FM/FFM, fat-free mass ratio; SPPB, Short Physical Performance Battery; 4MWT, 4-Meter Walk Test; STST, Sit-to-Stand test; HS, handgrip strength. (**A**) TCG, Tai Chi Group; (**B**) STG, Strength Training Group; (**C**) CG, Control Group. Percentage changes [∆% = (post–pre)/pre × 100] in parameters following a six-month intervention. * *p* < 0.01.

TCG and STG showed statistically significant percentage changes in physical activity reductions in time for STST (TCG −14%; STG −25%). Likewise, both groups showed a decrease in FM (%) (TCG and STG −9%); FM/FFM (TCG −15%; STG −14%) and an increase in SMM (TCG +9%; STG +8%) and SMMI (TCG +10%; STG +6%) (Figure 3).

### 3.5. Effect Size per Group (Partial Eta Squared, η_p_^2^)

Regarding the effect size, Table 5 presents the Partial Eta Squared estimates of the results that showed statistically significant differences in the ANCOVA (*p* < 0.01).

## 4. Discussion

Home-based tele-exercise (HBTE) is a viable and effective alternative for maintaining healthy physical activity in people who have limitations or difficulties performing it in community spaces outside the home [31,32]. In this sense, the confinement situation experienced during COVID-19 was a period that warranted the implementation of remote interventions for the control of chronic diseases and the promotion of healthy aging [33,34,35,36].

### 4.1. Dropout Rate and Reasons

Avoiding dropping to HBTE programs is the first challenge we face when implementing intervention programs in older adults, especially when the duration is several months. In this regard, a multicenter cohort study involving nine countries with a total sample of 760 people ≥18 years old who underwent an 8-week tele-intervention of physical exercise during the COVID-19 pandemic reported a dropout rate of 53.8%, highlighting among the main reasons the decrease in motivation to perform physical exercise, anxiety and the inconsistency in continuing with the measurements of the variables over time [37]. In our study, we observed around 50% in the total sample, with a slightly higher proportion in the TCG. The most frequent reasons were: (i) TCG, technical internet problems, difficulty using devices and lack of interest; (ii) STG, technical internet problems, difficulty using devices and need to return to work; and (iii) CG, difficulty using devices, technical internet problems and lack of interest. As can be seen, among the main reasons for dropping out in our study were technical internet problems and difficulty using devices, which should be taken into account for future research. In this regard, the Economic Commission for Latin America and the Caribbean (ECLAC) has reported and acknowledged a digital divide in Latin America, especially for older adults. Therefore, it is proposed to improve internet networks and develop training programs for older adults on the easy use of digital and smart devices [38]. This would allow for the promotion of remote physical exercise training as an option in situations of emergency confinement, such as the COVID-19 pandemic, or in situations where older adults have difficulty leaving their homes.

### 4.2. Nutrition

Although all three study groups were encouraged to adopt a healthy diet to maintain or improve muscle mass, including reduced consumption of carbohydrates and saturated fats, and increased intake of fruits and vegetables high in antioxidants, no statistically significant changes were observed between the groups after the intervention. In this regard, it has been shown that combined interventions, including physical exercise and adequate nutrition, are more effective than isolated programs of either physical exercise or nutrition [39]. Therefore, it is necessary to include precise, supervised guidelines to ensure significant dietary changes to maintain or increase skeletal muscle mass in old age, combined with HBTE.

### 4.3. Body Composition

Maintaining skeletal muscle mass and strength during the aging process is one of the challenges of healthy aging. Maintaining physical functional capacity allows older adults the mobility required to perform activities of daily living. In this regard, one of the best strategies is the adoption and maintenance of regular physical exercise programs, preferably supervised [40]. While resistance exercise is often cited as the most effective type of exercise for maintaining muscle mass in old age, the results are inconsistent. In our study, we found a statistically significant decrease in FM (%), along with an increase in SMM and SMMI in both experimental groups. These results are consistent with those reported in several studies [41,42]. However, the results contrast with the systematic review conducted by Chen et al. (2021), as they did not find statistically significant positive effects on SMM with physical exercise in the sum of 14 studies analyzed in healthy older adults [13]. Similarly, in the systematic review conducted by Yang et al. (2025), who reviewed 11 clinical trials of older adults with sarcopenia, no significant effects on SMMI were found [14]. In contrast, significant decreases in FM (%) have been reported in obese subjects who have undergone CT scans [43]. It is important to highlight the positive effects reported on skeletal muscle mass and muscle strength in the interventions carried out through digital health interventions in the systematic review of 11 clinical trials by Chen et al. (2025) [12].

The inconsistencies reported in several studies may be due to constitutional factors and the degree of muscle mass loss at the beginning of the study, in addition to intervening variables such as age, sex, diet, sleep, and gut microbiota, which are linked to the hallmarks of sarcopenia [44].

Another factor that must be considered in the variability of body composition results is the measurement method. In our study, body composition assessment was carried out through BIA with RJL System^®^, whose method is an appropriate tool for community-based screening when DXA is not feasible, as in the case of confinement due to the pandemic, enabling rapid, non-invasive, and low-cost estimation of lean and fat mass with acceptable agreement relative to DXA, thereby supporting early identification of body composition alterations associated with intrinsic capacity decline [45,46].

### 4.4. Physical Performance

Adequate physical performance reflects the strength, endurance, coordination, speed, and flexibility necessary to maintain optimal muscle capacity for continued independence. The SPPB test is often used to predict the risk of loss of independence and is a standard measure in research and clinical practice [47]. In this regard, Pavasini et al. (2016), through a systematic review and meta-analysis of 17 studies, demonstrated that an SPPB test score below 10 points is predictive of all-cause mortality, making maintaining this level throughout life beneficial for healthy aging [48].

Our findings showed no significant differences in the SPPB total score between groups. In this regard, only subtest STST(s) showed a statistically significant positive change with a decrease in time in both groups. This finding is consistent with what was previously published by our research group in a face-to-face community study [49].

### 4.5. Grip Strength

One of the most relevant physical changes associated with aging is the decrease in muscle strength, so increasing or maintaining it is a key objective for preventing or slowing the progression of sarcopenia in older adults. In our study, no statistically significant differences were observed in the analysis between groups. This may be due to the sample size, the quasi-experimental design of the study and the high dropout rate, since at the community level it has been shown that face-to-face physical effectiveness training is effective. In this regard, Labott et al. (2019), in a meta-analysis on the effect of strength and balance training on hand grip strength, which included twenty-four trials with a total of 3,018 participants (mean age 73.3 ± 6.0 years), reported a statistically significant effect (*p* < 0.001) [50]. Likewise, Wehner et al. (2021), in a meta-analysis on the effect of Tai Chi on grip strength in which twenty-one studies were included, found a statistically significant positive change [51]. These findings support the hypothesis that tele-training of exercise could improve or maintain grip strength. However, it is necessary to carry out more randomized clinical trials in confinement contexts to confirm the possible effect.

## 5. Strengths and Limitations

This intervention was the use of synchronous videoconferencing (Zoom^®^), which enabled real-time interaction between instructors and participants. Unlike asynchronous digital exercise programs, synchronous sessions allowed for immediate correction of movement execution, continuous monitoring of perceived exertion, rapid identification of potential safety issues, and direct social interaction among participants. These features may have contributed to maintaining motivation, improving adherence, and ensuring fidelity to the intervention. However, the success of the implementation also depended on participants having internet access, basic digital literacy, and adequate technological resources—factors that must be considered when transferring this intervention to other populations and contexts.

The main strength of the study is that it was conducted remotely via tele-exercise during an emergency lockdown in the context of the COVID-19 pandemic in a vulnerable population such as older adults. This unprecedented situation allowed for the quasi-experimental trial, albeit with many logistical and methodological limitations. Although 123 participants were initially recruited, only 65 completed the intervention and were included in the per-protocol analysis. Therefore, the effective statistical power was lower than anticipated, particularly for detecting small or moderate differences between groups. Consequently, non-significant results should be interpreted with caution.

Regarding these limitations, the sample was not representative; the assignment to the groups was not random, since the Control Group was made up of people who did not agree to participate in the exercise program, which led to selection bias. Furthermore, the percentage of participants who did not complete the study (refused the second measurement) was very high, although the reasons for this were unrelated to the intervention.

A relevant aspect to consider when interpreting these findings is the potential selection bias arising from the non-random assignment of participants to the Control Group. In this regard, it is possible that those who agreed to participate in the exercise interventions were more motivated to improve their health, more physically active, or more receptive to the behavioral interventions than those who declined them. While no significant baseline differences were observed in the variables assessed, it is possible that differences in unmeasured psychosocial, behavioral, or socioeconomic factors influenced the intervention results. Furthermore, the influence of prior physical exercise history within the six months established in the inclusion criteria was not considered. Additionally, employment status was not uniform, leading some participants to drop out of the study to return to their jobs.

## 6. Conclusions

Our findings suggest that tele-training in Tai Chi or strength exercises could have a positive effect on skeletal muscle mass, along with a decrease in body fat percentage compared to the Control Group. Therefore, this type of intervention could be an option for older adults to promote healthy lifestyles, such as maintaining physical activity and avoiding social isolation during lockdowns like the one experienced during the COVID-19 pandemic. However, the results cannot be generalized due to limitations in the quasi-experimental study design, the non-representative sample size, the high dropout rate, and selection bias. Therefore, intention-to-treat randomized clinical trials with representative samples and tele-exercise programs for older adults in confinement situations are needed.

## Figures and Tables

**Table 1:** Sociodemographic characteristics and health status by study group.

Variable	TCG n = 22	STG n = 22	CG n = 21	*p*-Value *
Age (years)	65 ± 4	64 ± 3	66 ± 4	0.15
Sex, Female (%)	19 (86)	19 (86)	14 (64)	0.15
Schooling (years)	11 ± 5	14 ± 6 *	10 ± 5	0.02
Living with (%)	16 (73)	18 (82)	16 (76)	0.70
Number of people they live with	4 ± 3 *	2 ± 1.86	2 ± 1.83	0.03
Economic income ($)	6000 (3000–8000)	10,000 (5000–18250)	5694 (2000–8000)	0.016
Diabetes Mellitus Type 2 n (%)	8 (36)	3 (14)	2 (9)	0.04
SAH n (%)	12 (55)	7 (30)	9 (41)	0.26
Joint disorder n (%)	4 ( 20)	7 (30)	4 (18)	0.58
Heart disease n (%)	1 (5)	0 (0)	2 (9)	0.34
Polypharmacy n (%)	1 (5)	4 (17)	5 (23)	0.17
SBP mm Hg	138 ± 16	123 ± 18 *	143 ± 20	0.03
DBP mm Hg	85 ± 12	77 ± 9 *	80 ± 8	0.03

Abbreviations: TCG, Tai Chi Group; STG, Strength Training Group; CG, Control Group; ($) Mexican pesos; SAH: Systemic Arterial Hypertension, SBP: Systolic Blood Pressure, DBP: Diastolic Blood Pressure, ANOVA one-way * *p* < 0.05.

**Table 2:** Nutrient intake by study group.

Nutrient	TCG n = 22	STG n = 22	CG n = 21	*p*-Value
Energy (kcal)				
Basal	1624 (1316–2205)	1736 (1359–2378)	1723 (1415–2254)	
6 months	1760 (1268–2485)	1587 (1170–1926)	1418 (1077–1796)	
Effect (∆)	136 (−396–491)	−150 (−509–465)	−306 (−731–114)	0.45
Protein (g/kg) *				
Basal	0.96 (0.73–1.09)	0.97 (0.89–1.26)	1.12 (0.80–1.38)	
6 months	0.99 (0.86–1.26)	0.90 (0.74–1.17)	0.89 (0.73–1.46)	
Effect (∆)	0.15 (−0.13–0.27)	−0.18 (−0.46–0.18)	−0.08 (−0.49–0.57)	0.56
Carbohydrates (g)				
Basal	238 (169–294)	230 (175–296)	250 (182–336)	
6 months	263 (176–340)	210 (160–245)	183 (151–251)	
Effect (∆)	21 (−60–94)	−39 (−79–80)	−62 (−151–12)	0.26
Fat (g)				
Basal	54 (34–95)	55 (46–67)	52 (44–70)	
6 months	49 (31–74)	50 (39–71)	44 (31–72)	
Effect (∆)	−4 (−21–14)	−11 (−28–7)	−7 (−2–14)	0.60
Fiber (g)				
Basal	22 (13–34)	26 (16–36)	24 (14–31)	
6 months	26 (19–32)	23 (17–32)	16 (13–29)	
Effect (∆)	7 (−2.2–14)	1 (−11–10)	2 (−14–8)	0.46
Sugar (g)				
Basal	73 (43–105)	65 (48–93)	94 (66–130)	
6 months	80 (68–123)	66 (52–84)	69 (48–99)	
Effect (∆)	16 (−16–59)	−8 (−41–27)	−18 (−50–13)	0.09

Abbreviations: TCG, Tai Chi Group; STG, Strength Training Group; CG, Control Group. Friedman and Kruskal–Wallis; effect (∆), the difference in changes before and after the intervention. *p*-value, between groups. * (g/kg), total protein intake relative to body weight.

**Table 3:** Body composition changes by study group.

Variable	TCG n = 22	STG n = 22	CG n = 21	*p*-Value
Weight (Kg)				
Basal	74 ± 3.23	75 ± 2.75	75 ± 3.91	0.79
6 months	72 ± 3.10	73 ± 3.65	73 ± 3.75	
Mean difference (95% CI)	−1.72 (−3.07, −0.37)	−2.33 (−3.49, −1.18)	−2.10 (−3.73, −0.47)	
BMI (Kg/m^2^)				
Basal	30.08 ± 2.24	29.85 ± 2.06	31.1 ± 2.50	0.76
6 months	29.37 ± 2.17	28.85 ± 2.03	30.4 ± 2.42	
Mean difference (95% CI)	−0.87 (−1.29, −0.22)	−0.99 (−1.48, −0.51)	−0.84 (−1.53, −0.15)	
SMM (kg)				
Basal	18.82 ± 0.67	20.22 ± 0.57	19.40 ± 0.78	<0.01
6 months	20.62 ± 0.78	21.32 ± 0.66	18.52 ± 0.91	
Mean difference (95% CI)	1.79 (0.71, 2.88) *	1.10 (0.18, 2.02) *	−0.88 (−2.15, 0.38)	
SMMI (Kg/m^2^)				
Basal	7.68 ± 0.25	7.92 ± 0.22	8.05 ± 0.30	<0.01
6 months	8.42 ± 0.27	8.30 ± 0.23	7.68 ± 0.31	
Mean difference (95% CI)	0.73 (0.28, 1.19) *	0.40 (0.05, 0.77) *	−0.37 (−0.89, 0.15)	
PA (°)				
Basal	5.93 ± 0.23	5.47 ± 0.19	5.83 ± 0.55	0.09
6 months	5.77 ± 0.42	6.36 ± 0.35	5.19 ± 0.75	
Mean difference (95% CI)	0.15 (−0.54, 0.49)	0.79 (0.28, 1.69)	−0.64 (−1.74, 0.46)	
FM (%)				
Basal	47.74 ± 1.54	45.83 ± 1.31	46.59 ± 1.80	<0.01
6 months	42.93 ± 1.71	41.84 ± 1.45	49.48 ± 2.00	
Mean difference (95% CI)	−4.81 (−8.17, −1.44) *	−3.99 (−6.84, −1.13) *	2.89 (−1.03, 6.81)	
FFM (Kg)				
Basal	37.87 ± 1.65	40.29 ± 1.40	40.67 ± 1.93	0.11
6 months	38.06 ± 1.71	42.16 ± 1.45	38.51 ± 2.00	
Mean difference (95% CI)	1.89 (0.22, 3.85)	2.03 (0.12, 3.94)	−2.28 (−4.81, 0.35)	
FM/FFM				
Basal	0.95 ± 0.05	0.88 ± 0.04	0.94 ± 0.05	<0.01
6 months	0.81 ± 0.05	0.75 ± 0.04	1.00 ± 0.06	
Mean difference (95% CI)	−0.14 (−0.23, −0.54) *	−0.13 (−0.20, −0.05) *	0.06 (−0.03, 0.17)	

Abbreviations: TCG, Tai Chi Group; STG, Strength Training Group; CG, Control Group. BMI, Body Mass Index; SMM, skeletal muscle mass; SMMI, Skeletal Muscle Mass Index; PA, phase angle; FM (%), percentage of fat mass; FFM, fat-free mass; FM/FFM ratio. CI: confidence interval. ANCOVA adjusted by Diabetes Mellitus Type 2, sex, years of schooling, number of people living with, SBP and DBP. * *p*-value < 0.05, post hoc MD between-groups CTG vs. CG and STG vs. CG. The MD of all variables between-groups CTG vs. STG were not statistically significant (*p* > 0.05).

**Table 4:** Changes in physical performance and grip strength by study group.

Variable	TCG n = 22	STG n = 22	CG n = 21	*p*-Value
SPPB (Score)				
Basal	9.13 ± 0.33	10.25 ± 0.30	10.07 ± 0.42	0.43
6 months	9.99 ± 0.47	10.37 ± 0.42	10.06 ± 0.59	
Mean difference (95% CI)	0.86 (−0.10, 1.83)	0.18 (−0.75, 0.97)	−0.01 (−1.22, 1.21)	
4MWT(s)				
Basal	1.05 ± 0.29	0.75 ± 0.17	0.81 ± 0.19	0.09
6 months	0.95 ± 0.3	0.77 ± 0.20	0.79 ± 0.17	
Mean difference (95% CI)	−0.13 (−0.28, 0.13)	0.05 (−0.08, 0.18)	0.22 (0.02, 0.41)	
STST(s)				
Basal	15.86 ± 0.96	14.28 ± 0.81	12.54 ± 1.19	<0.01
6 months	11.39 ± 0.87	12.26 ± 0.73	13.75 ± 1.07	
Mean difference (95% CI)	−4.47 (−6.90, −2.04) *	−2.02 (−4.07, −0.04) *	1.21 (−1.78, 4.20)	
HS (Kg)				
Basal	23.64 ± 1.35	26.00 ± 1.16	24.75 ± 1.63	0.44
6 months	22.69 ± 1.60	26.64 ± 1.39	23.20 ± 1.94	
Mean difference (95% CI)	−0.09 (−3.32, 1.44)	0.63 (−1.04, 2.69)	−1.55 (−4.41, 1.31)	

Abbreviations: TCG, Tai Chi Group; STG, Strength Training Group; CG, Control Group. SPPB: Short Physical Performance Battery; 4MWT(s), 4-Meter Walk Test; STST(s), Sit-to-Stand test; HS (Kg) (handgrip strength); CI: confidence interval. ANCOVA adjusted by Type 2 Diabetes Mellitus, sex, years of schooling, number of people living with, SBP and DBP. * *p*-value < 0.05, post hoc MD between-groups CTG vs. CG and STG vs. CG. The MD of all variables between-groups CTG vs. STG were not statistically significant (*p* > 0.05).

**Table 5:** Effect size estimated by Eta Squared.

Variable	F	*p*-Value	Partial Eta Squared
Weight (Kg)	0.22	0.79	0.10
BMI (Kg/m^2^)	0.27	0.76	0.01
SMM (kg)	5.38	0.008 *	0.19
SMMI (Kg/m^2^)	5.23	0.009 *	0.19
PA (°)	2.51	0.09	0.10
FM (%)	5.06	0.01 *	0.18
FFM (Kg)	2.02	0.11	0.15
FM/FFM	5.47	0.008 *	0.19
SPPB (Score)	0.85	0.43	0.04
4MWT(s)	4.57	0.09	0.17
STST(s)	4.42	0.01 *	0.16
HS (Kg)	0.83	0.44	0.03

Abbreviation: BMI, Body Mass Index; SMM, skeletal muscle mass; SMMI, Skeletal Muscle Mass Index; PA, phase angle; FM (%), percentage of fat mass; FFM, fat-free mass; FM/FFM ratio. SPPB: Short Physical Performance Battery; 4MWT(s), 4-Meter Walk Test; STST(s), Sit-to-Stand test; HS (Kg) (handgrip strength). ANCOVA * *p* significant < 0.01 between-group effect size. A partial η2 Eta value close to ≥0.01 is considered low, ≥0.06 medium, and a value greater than ≥0.14 large.

## Data Availability

Data presented in this study are available on request from the corresponding author. Our university states that research results information can only be provided when formally requested from the project manager.

## Supplementary Materials

The following supporting information can be downloaded at: https://www.mdpi.com/article/10.3390/sports14090372/s1. Table S1: Description of Tai Chi training sessions. Table S2: Description of strength training sessions.

## Abbreviations

The following abbreviations are used in this manuscript:

TCG	Tai Chi Group
STG	Strength Training Group
CG	Control Group
($)	Mexican Pesos
SAH	Systemic Arterial Hypertension
SBP	Systolic Blood Pressure
DBP	Diastolic Blood Pressure
BMI	Body Mass Index
SMM	Skeletal Muscle Mass
SMMI	Skeletal Muscle Mass Index
PA	Phase Angle
FM (%)	Fat Mass Percentage
FFM	Fat-Free Mass
SPPB	Short Physical Performance Battery
4MWT(s)	4-Meter Walk Test
STST(s)	Sit-to-Stand Test
