# Supplementary material for: Effect of Remote Tai Chi Training vs. Strength Training on Muscle Mass and Grip Strength in Older Mexican Adults in the Context of the COVID-19 Pandemic
Source: Sports (Basel). 2026 Sep 1;14(9):372. doi: 10.3390/sports14090372 (PMC13611292; doi:10.3390/sports14090372)
Supplement: Supplementary file 1 [file sports-14-00372-s001.zip › sports-4342720-supplementary.pdf]

Table S1. Supplementary Materials S1. Description of Tai Chi training sessions.

**Tai Chi**	
Frequency: 4 days for week	Intensity: Borg scale, light to moderate
Time for session: 60 minutes	Materials: Comfortable clothing, athletic shoes, and hydration.
**Warm Up**<br><br>Duration: 10 minutes<br><br>Head-to-toe movements for joint lubrication:<br><br>• Forward and backward neck flexion.<br>• Side neck flexion.<br>• Side neck rotation.<br>• Right head circles.<br>• Left head circles.<br>• Shoulder circles forward, arms hanging at the sides.<br>• Shoulder circles backward, arms hanging at the sides.<br>• Crossing arms in front of the chest.<br>• Alternating arm movements forward and backward.<br>• Circular arm movements forward.<br>• Circular arm movements backward.<br>• Hip circles to the left.<br>• Hip circles to the right.<br>• Hip flexion forward, touching the floor with hands.<br>• Alternating leg raises with legs extended.<br>• Alternating leg raises, knees bent.<br>• Alternating leg raises, heels bent.<br>• Alternating lateral leg raises with legs extended.<br>• Rise up onto your toes.<br>• Circles with your right ankle.<br>• Circles with your left ankle.<br>• March in place.<br>• Jog in place.<br>• Heel-to-buttock touch.<br>• Jog in place.<br>• Hip flexion touch to touch your toes, knees, hips, and shoulders with your hands.<br>• Jog in place.<br>• Side leg swings.<br>• Jog in place.<br>• Heel-to-toe touch.<br><br>**10 to 15 seconds for movement, making variations per week.**	

**Tai Chi Yang**<br><br>Duration: 40 minutes<br><br>• Starting Stance<br>• Left Deflection<br>• Right Deflection<br>• Roll<br>• Starting Stance<br>• Left Deflection<br>• Right Deflection<br>• Roll<br>• Press<br>• Double Elbow<br>• Single Whip<br>• Raise Hands<br>• The Rooster Stands on One Leg<br>• The Snake Slithers<br>• Mount the Tiger<br>• Draw the Bow<br>• Shoot the Tiger
**Stretching** of body segments while maintaining a static position and guided relaxation.<br><br>Duration: 10 minutes.

Table S2. Supplementary Materials S2. Description of strength training sessions.

**Entrenamiento de fuerza**	
Frequency: 4 days a week	Intensity: Borg scale, light to moderate
Time for session: 60 minutes	Materials: Mat, chair, Theraband resistance bands, dumbbells. Comfortable clothing, athletic shoes, and water.
**Warm Up**<br><br>Duration: 10 minutes<br><br>Head-to-toe movements for joint lubrication:<br><br>• Forward and backward neck flexion.<br>• Side neck flexion.<br>• Side neck rotation.<br>• Right head circles.<br>• Left head circles.<br>• Shoulder circles forward, arms hanging at the sides.	

- Shoulder circles backward, arms hanging at the sides.
- Crossing arms in front of the chest.
- Alternating arm movements forward and backward.
- Circular arm movements forward.
- Circular arm movements backward.
- Hip circles to the left.
- Hip circles to the right.
- Hip flexion forward, touching the floor with hands.
- Alternating leg raises with legs extended.
- Alternating leg raises, knees bent.
- Alternating leg raises, heels bent.
- Alternating lateral leg raises with legs extended.
- Rise up onto your toes.
- Circles with your right ankle.
- Circles with your left ankle.
- March in place.
- Jog in place.
- Heel-to-buttock touch.
- Jog in place.
- Hip flexion touch to touch your toes, knees, hips, and shoulders with your hands.
- Jog in place.
- Side leg swings.
- Jog in place.
- Heel-to-toe touch.

10 to 15 seconds for movement, making variations per week.

Strength training

Duration: 40 minutes with progressive overload.

Months 1-3: Basic loads, bodyweight exercises for two body segments with 3 cycles each, consisting of 3 sets of 8-12 repetitions with a 1-minute rest between each set.

Months 3-6: Progressive loads with resistance bands (Theraband) and dumbbells for two body segments with 3 cycles each, consisting of 3 sets of 8-12 repetitions with a 1-minute rest between each set.

- Wall push-up.
- Wall walk.
- Bicep curl.
- Lateral extension of arms and torso.
- Half push-up.
- Boxer's punch, with band, with dumbbell.
- Front raise of arms, with band, with dumbbells.

• Shoulder press with band.<br>• Chest press with band.<br>• Tricep extension with band.<br>• Half push-up.<br>• Lunge, starting alone, then with band, and finally with dumbbell.<br>• Squat with chair support, progressing to standing, then with band, and finally with band and dumbbell.
**Stretching** by body segments while maintaining a static position and guided relaxation.<br><br>Duración 10 minutos.
